# Coexistent Circumvallate Placenta and Battledore Insertion of Umbilical Cord Resulting in Grave Obstetric Outcome: A Case Report

**Published:** 2017

**Authors:** Nalini Sharma, Rituparna Das, Sushila Salam, Roma Jethani, Ahanthem Santa Singh

**Affiliations:** Department of Obstetrics and Gynaecology, North Eastern Indira Gandhi Regional Institute of Health and Medical Sciences, Shillong, Meghalaya, India

**Keywords:** Battledore insertion of umbilical cord, Circumvallate placenta, Preeclampsia, Pregnancy outcome

## Abstract

**Background:**

Various placental and cord abnormalities have been known to adversely affect the obstetric outcome. Circumvallate placenta and Battledore insertion of the umbilical cord are both rare and known to be associated with poor obstetric outcome individually.

**Case Presentation:**

In this case report, the woman presented at 8 months of gestation with preeclampsia with IUFD to North Easter Indira Gandhi Regional Institute of Health and Medical Science Shillong on 22/7/16 and delivered a macerated fetus vaginally. After delivery, examination revealed both a circumvallate placenta and Battledore insertion of umbilical cord. This might have attributed to preeclampsia and ultimately IUFD in this case as she had no other identifiable cause for IUFD.

**Conclusion:**

If such placental and cord abnormalities are suspected or diagnosed antenatally by ultrasonography, the pregnancy should be regarded as high risk. Such woman would require more stringent follow up in the antenatal period and continuous intrapartum monitoring to avoid any catastrophe and to achieve a favorable maternal and fetal outcome.

## Introduction

Various placental and cord abnormalities have been known to adversely affect the obstetric outcome. Circumvallate placenta is an abnormality of the placental shape where there is a central depression on the foetal surface surrounded by a greyish white thickened ring at the periphery (1). Here the chorion and amnion are folded upon themselves at the placental margin making the chorionic plate on the foetal side smaller than the basal plate on the maternal side leaving a part of the placenta uncovered at the periphery (1). In Battledore placenta, the umbilical cord is attached to the placental margin (1) also known as marginal insertion of umbilical cord. Both abnormalities are very rare individually; the prevalence of circumvallate placenta is not documented which could be because of its rarity; however, prevalence of Battledore insertion of umbilical cord is reported as 7% in term pregnancies (2). Circumvallate placenta is known to be associated with late abortions, preterm deliveries, abruptio placentae, foetal growth restriction, non-reassuring foetal heart rate patterns, increased perinatal mortality and congenital malformations (1, 3–5). Association of circumvallate placenta and preeclampsia is not very clearly mentioned. Battledore insertion of the umbilical cord is though reported to have little clinical significance by some studies (6, 7), others have reported increased incidence of placenta previa, abruptio placentae, preeclampsia, preterm delivery, increased incidence of emergency LSCS and poor neonatal outcome (8, 9). Combination of circumvallate placenta with Battledore insertion of the cord has been reported only in one study (10). This case has been reported as the women had a coexistence of both these rare abnormalities with a rather grave prognosis of intrauterine foetal demise.

## Case Presentation

A 35 year old lady G5P4L4, with all previous normal deliveries, referred from a district hospital, attended the Emergency Department of North Easter Indira Gandhi Regional Institute of Health and Medical Science Shillong on 22/02/2017 at 8 months of pregnancy with absent foetal movements since the last 2 days. She could not recall her LMP. She had no significant past medical or surgical history. On general examination, she was not pale, icterus, oedema, cyanosis and dehydration were all absent, temperature was 98^0^F, pulse rate 88/*min* and B.P was 160/90 *mmHg*. The CNS, CVS and respiratory tract examination were within normal limits. On obstetric examination, fundal height corresponded to 34 weeks of gestation, uterine tone was normal and fetus in cephalic presentation and fetal heart sound could not be localized. Pervaginal examination revealed that the cervix was 1.5 *cm* dilated and 50% effaced with intact membranes and adequate pelvis. Her urine albumin was 2+ on dipstick. All relevant investigations were sent and IUFD was confirmed by ultrasound and there was no congenital malformation in the fetus by ultrasound. Labor induction was done. Her blood group was B positive, Hb 10.6 *g/dl*, TLC 10,600/cumm, DLC N76L16 M6E2, platelet count was 3.3 lacs/cumm, RBS 77 *mg/dl*, LFT-Total bilirubin 0.7 *mg/dl*, SGOT 23 *U/L*, SGPT 24 *U/L*, total protein 6.2 *mg/dl*, blood urea 28 *mg/dl*, Sr. creatinine 1.0 *mg/dl*, Prothrombin time 15 secs, APTT 40.1 secs, INR 1.15, VDRL nonreactive, HbsAg negative, HIV nonreactive. She delivered vaginally a macerated female baby of 1.8 *kg*. Active management of the third stage of labor was done and thereafter labor events were uneventful.

**Figure 1.**
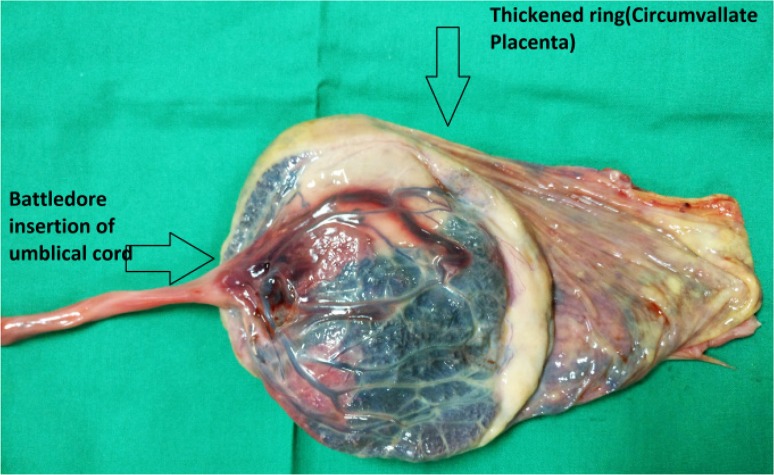
Coexistent Battledore insertion of umbilical cord and circumvallate placenta

On clinical examination after delivery, the baby did not have any gross congenital malformation. Liquor was absent and no meconium stain was seen. Examination of the placenta revealed a circumvallate placenta with Battledore insertion of the cord (Figure 1). There was no retro placental clot and the umbilical cord was normal in length without any knots. No abnormality was detected in the membranes. Autopsy of baby was advised but the patient and her attendance were not willing for autopsy.

Postpartum period was uneventful and she was discharged in good health on the second day of puerperium.

Histopathological examination of the placenta reported the cord as having two arteries and one vein which were patent and within normal limits and sections from placenta showed large areas of haemorrhagic infarction. Focal areas of chorangiosis suggestive of chronic hypoxia were also noted.

## Discussion

In the past as stated by Williams, circumvallate placenta was considered to be a mere interesting anatomic variation of placenta without any clinical significance (3). Since then, various studies have been conducted and in one of the earlier studies by Russell et al., high incidence of late abortions, preterm labors and maternal haemorrhage have been found to be associated with circumvallate placenta. Although high incidence of foetal demise was associated with circumvallate placenta, it was rarely listed as the cause (3).

Subsequently, other studies have also shown increased incidence of complications in patients with circumvallate placenta like abruptio placentae, preterm births, IUGR, oligohydramnios, non-reassuring foetal heart rate patterns (4, 5) and even a study reported increased incidence of IUFD as compared to controls (5). Also, in addition to these complications, Hanako Taniguchi et al. stated that circumvallate placenta was associated with increased incidence of emergency Caesarean Section, NICU admission, neonatal death and even chronic lung disease (6).

Battledore insertion of cord was also found to have increased association with placenta previa, abruptio placentae, preeclampsia, preterm delivery, cord prolapse, foetal distress, increased incidence of emergency LSCS, low Apgar score, NICU admission, low birth weight and congenital malformations (8, 9). Keeping in view that this patient had an IUFD without any identifiable cause for the same, it may be suspected that the coexistence of both these abnormalities might have attributed to preeclampsia and ultimately the grave prognosis of IUFD.

The diagnosis of circumvallate placenta antenatally is however very difficult. The accuracy of diagnosis by ultrasound is limited with high false positive and high false negative rates (11). Second trimester bleeding per vaginum and premature rupture of membranes had a sensitivity of 28.8% and specificity of 99.9% when both are used as predictors for circumvallate placenta (6). Also, the diagnosis of Battledore placenta by ultrasound is possible and is better done in second trimester as localization of the site of cord insertion becomes difficult with increasing gestational age (12). Women with such abnormalities of placental and cord if detected antenatally should be considered as high risk pregnancy due to possible poor pregnancy outcome.

## Conclusion

Both circumvallate placenta and Battledore insertion of the umbilical cord are rare and are known to adversely affect the pregnancy outcome individually and in these women, their coexistence leads to a more serious untoward consequence of IUFD. Thus, if such placental and cord abnormalities are suspected or diagnosed antennatally by ultrasonography, the pregnancy should be regarded as high risk and the women would require more stringent follow up in the antenatal period and continuous intrapartum monitoring to avoid such a catastrophe and to achieve favorable maternal and foetal outcome.
